# Safety and Efficacy of Combined Coronary and Peripheral Percutaneous Revascularization: A Proof-of-Concept Study

**DOI:** 10.3390/jcm13154516

**Published:** 2024-08-02

**Authors:** Mario Enrico Canonico, Nicola Verde, Marisa Avvedimento, Attilio Leone, Maria Cutillo, Fiorenzo Simonetti, Salvatore Esposito, Luca Bardi, Giuseppe Giugliano, Eugenio Stabile, Raffaele Piccolo, Giovanni Esposito

**Affiliations:** 1 Department of Advanced Biomedical Sciences, University of Naples Federico II, 80131 Naples, Italy; mecanonico@me.com (M.E.C.);; 2 CPC Clinical Research, University of Colorado, Aurora, CO 80045, USA; 3 Quebec Heart and Lung Institute, Laval University, Quebec City, QC G1V0A6, Canada; 4 San Giuseppe Moscati Hospital, 83100 Avellino, Italy; 5 Italian National Institute of Health, 00161 Rome, Italy; 6 Division of Cardiology, Cardiovascular Department, Azienda Ospedaliera Regionale “San Carlo”, 85100 Potenza, Italy

**Keywords:** lower extremity artery disease, percutaneous coronary intervention, concomitant revascularization, peripheral artery disease, multivascular disease

## Abstract

**Background**. Lower extremity peripheral artery disease (LEPAD) frequently coexists with coronary artery disease (CAD) in patients with multisite vascular disease (MVD). While percutaneous revascularization is well-established for both LEPAD and CAD, limited evidence exists for patients eligible for both procedures. Specifically, the feasibility of concomitant LEPAD and CAD percutaneous revascularization remains unknown. **Objectives**. To compare the efficacy and safety of concomitant coronary and lower extremity elective percutaneous revascularization. **Methods**. Between 2012 and 2021, we included 135 patients in an observational, retrospective single-center registry. The population was stratified into two groups: 45 patients (concomitant group) underwent simultaneous coronary and peripheral percutaneous interventions, and 90 patients (deferred group) underwent two separate procedures within one year. The primary efficacy endpoint was major adverse cardiovascular events (MACE) at one year, while the primary safety endpoint was in-hospital contrast-induced nephropathy (CIN). **Results**. Study groups were well-balanced in baseline characteristics. In terms of coronary features, the concomitant revascularization group more often underwent single-vessel percutaneous coronary intervention (PCI), while the deferred group had multivessel PCI with diffuse coronary disease. No differences were detected in the number of LEPAD lesions between groups. For the primary efficacy endpoint, the incidence of MACE at one year was 37.8% in the concomitant group vs. 34.4% in the deferred group (HR 1.20, 95% CI 0.64–2.10; *p* = 0.61). No significant differences were found in CIN occurrence between the concomitant and deferred groups (11.1% vs. 8.9%; OR 1.30; 95% CI 0.36–4.21; *p* = 0.68). **Conclusions**. Multisite vascular disease patients eligible for CAD and LEPAD percutaneous revascularization exhibited a high cardiovascular risk profile with diffuse multivessel coronary and lower extremity disease. Our study suggests the efficacy and safety of concomitant coronary and lower extremity percutaneous revascularization based on one-year MACE incidence and in-hospital CIN. However, dedicated studies are warranted to confirm the short- and long-term outcomes of the concomitant revascularization strategy.

## 1. Introduction

Atherosclerotic cardiovascular disease (ASCVD) is a leading cause of major adverse cardiovascular events (MACE) and major adverse limb events (MALE) [1]. Advanced ASCVD affects multiple vascular districts, with prevalence ranging from 25 to 70% for coronary artery disease (CAD) and 14–19% for carotid stenosis in patients affected by lower extremity peripheral artery disease (LEPAD) [1].

Irrespective of limb symptoms, LEPAD patients show an increased risk of overall death, cardiovascular (CV) death, fatal myocardial infarction (MI), and stroke [2]. The risk of ischemic events is higher in patients with polyvascular disease compared to those without [3]. Data from the VOYAGER-PAD trial confirmed the higher risk of MACE plus MALE in patients with CAD compared to those without in a primary PAD population underwent lower extremity revascularization (LER) [4].

Despite the known prognostic value of concomitant CAD and LEPAD, established recommendations for coronary disease screening in lower extremity disease patients suitable for endovascular revascularization, or vice versa, are lacking. This gap in evidence hinders the clarification of the risk-benefit balance between concomitant and deferred percutaneous revascularization for elective patients eligible for CAD and LEPAD interventions.

The aim of our study was to assess the safety and effectiveness of concomitant vs. deferred CAD and LEPAD percutaneous revascularization in a cohort of LEPAD patients with multisite vascular disease.

## 2. Methods

### 2.1. Patient Population and Data Collection

Consecutive patients with concomitant CAD and LEPAD undergoing elective coronary and lower extremity arterial percutaneous revascularization between 2012 and 2021 at our institution were included in the study. Patients requiring urgent/primary percutaneous coronary intervention (PCI) or urgent percutaneous transluminal angioplasty (PTA) were excluded. A full list of inclusion and exclusion criteria is listed in Table 1. Clinical, procedural, and follow-up data were anonymously entered in a web-based database (https://www.redcap.unina.it/redcap/, 1 March 2022). The present study has been approved by the local ethic committee, and all study-related procedures were carried out in accordance with the Declaration of Helsinki. Written informed consent was obtained for all patients included in the study.

### 2.2. Indication to Procedure

According to current American College of Cardiology/American Heart Association (ACC/AHA) and European Society of Cardiology (ESC) guidelines, LEPAD was defined as the finding of a value ≤ 0.9 of ankle-brachial index (ABI) [5]. In patients with a high clinical suspicion of LEPAD despite an ABI > 0.9, the first line imaging method to confirm disease was duplex ultrasound. Moreover, patients with life-limiting symptoms despite optimized pharmacological therapy (defined at least as an IIB class according to Fontaine classification) and/or with a hemodynamically significant lesion of aorto-iliac, femoral-popliteal, or below the knee district were considered suitable for percutaneous revascularization and included in the study [1,5].

Significant CAD was defined as the finding of an ischemic coronary lesion documented by an abnormal stress-electrocardiogram or stress-echocardiography, an ischemic area > 10% of the left ventricle documented by myocardial single photon emission computed tomography (SPECT) or positron emission tomography (PET), a significant stenosis of at least one vessel detected by coronary computed tomography, a coronary stenosis > 70% in a non-left main vessel and >50% in the left main without a non-invasive or invasive ischemic probe, an intermediate stenosis in a non-left main vessel without a non-invasive ischemia probe but with a fractional flow reserve (FFR) value ≤ 0.8 [6,7]. The choice of concomitant vs. deferred coronary and LEPAD percutaneous was left to operator discretion.

The institution where the study was performed includes a 24/7 service of cardiac and vascular surgery. The catheterization laboratory includes 3 cardiologists with high and recognized experience in coronary and peripheral revascularization.

### 2.3. Clinical Follow-Up and Study Endpoints

After hospital discharge, follow-up was performed at 30-day intervals and after 1 year through clinical visits, telephone contacts, electronic medical records, or by formal inquiries to primary care physicians. All adverse events were systematically recorded using a web-based reporting system and classified according to the following definitions.

The primary efficacy endpoint of the study was the incidence of MACE, defined as the composite of spontaneous myocardial infarction, death for all causes, any percutaneous transluminal angioplasty, any percutaneous coronary intervention, any amputation for vascular causes, or ischemic stroke at 30-day follow-up and up to 1 year after the last intervention. Secondary endpoints included in-hospital vascular access complications, periprocedural MI, and periprocedural myocardial injury. The primary safety endpoint was in-hospital contrast-induced nephropathy (CIN). Major adverse limb events (MALE) were defined as any percutaneous transluminal angioplasty or any amputation for vascular causes. The incidence of vascular complications was assessed by duplex ultrasound during hospitalization, while the laboratory data were collected at baseline and after 24–48 h from the procedures (i.e., hemoglobin, Hs-TnI, CK-MB, and creatinine). Vascular access complications were defined as arterial injury (i.e., perforation, rupture, dissection, arteriovenous fistula, pseudoaneurysm, hematoma, retroperitoneal hematoma, infection) or compartment syndrome resulting in death, Bleeding Academic Research Consortium (BARC) type ≥ 3a bleeding [8,9].

Periprocedural, spontaneous MI, and myocardial injury were assessed according to the fourth universal definition (UDMI) based on rise/fall of cardiac troponin I (cTnI) values above the 99th percentile Upper Reference Limit (URL) with or without (as myocardial injury) at least one between symptoms of myocardial ischaemia, new ischaemic ECG changes, development of pathological Q waves, imaging evidence of new loss of viable myocardium, or new regional wall motion abnormality in a pattern consistent with an ischaemic aetiology or identification of a coronary thrombus by angiography or autopsy [10].

Chronic kidney disease (CKD) was considered as an estimated glomerulation filtration rate (eGFR) < 60 mL/min/1.73 m^2^ assessed by the Modification of Diet in Renal Disease (MDRD) equation, and CIN was defined as an increase in serum creatinine by ≥0.3 mg/dL (≥26.5 μmol/L) within 48 h [11].

Stroke was defined as an episode of neurological dysfunction caused by focal cerebral, spinal, or retinal infarction [12]. Any PTA and any PCI were both considered as peripheral or coronary revascularization within 1 year from the index one of the same or another lesion. Amputation due to a vascular etiology was defined as above (major) or below (minor) the ankle for non-traumatic causes and due to a vascular cause, including worsening perfusion of the limb as the primary indication, with the exclusion of infectious causes [13].

### 2.4. Statistical Analysis

Continuous data distribution was assessed with the Kolmogorov–Smirnov test. Normally distributed variables are expressed as mean ± standard deviation, while non-normally distributed ones are expressed as median and interquartile range. Categorical variables are expressed as numbers and percentages. Continuous normally distributed variables were compared by using the unpaired t test, and differences between non-normally distributed variables were assessed with the Mann–Whitney U test. Categorical variables were assessed using the Fisher’s exact test. A *p* value of <0.05 was considered to indicate a statistically significant difference for all the analyses performed. A logistic regression model was used to test for between-group differences with adjustment for clinical outcomes (cardiac arrest, fourth UDMI Periprocedural MI, fourth UDMI myocardial injury, contrast-induced nephropathy) at the baseline. The estimates from the model are expressed in terms of odds ratios (OR).

In addition, a Kaplan–Meier analysis was performed to determine survival cumulative probabilities for time-to-event outcomes, including six MACE: death for all causes, any amputation for vascular causes, spontaneous MI, any PTA, any PCI, or ischemic stroke. Cox proportional hazard models were used to investigate the association of concomitant PTA at the time of PCI with risk of clinical outcomes and were estimated using the HRs and their 95% confidence intervals (CI). Time to response was displayed by a Kaplan–Meier plot. Since the retrospective nature of this proof-of-concept study, no formal assessment for sample size was performed. All data analyses were performed using the R version 4.1.3 statistical software.

## 3. Results

### 3.1. Baseline Characteristics

**Figure 1:**
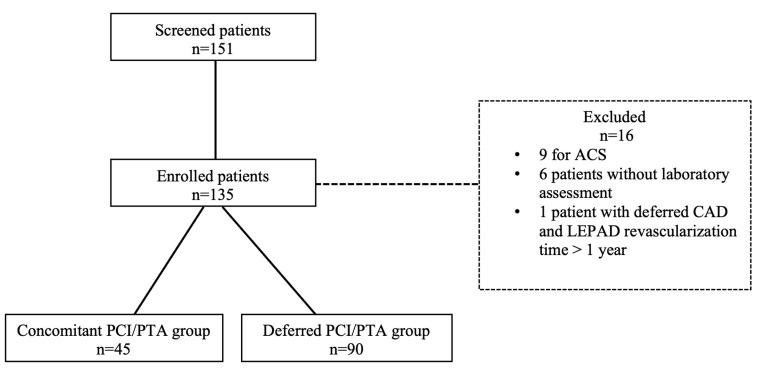
Study flowchart. ACS: acute coronary syndrome; CAD: coronary artery disease; LEPAD: lower extremity peripheral artery disease; PCI: percutaneous coronary intervention; PTA: percutaneous transluminal angioplasty.

Among 151 screened patients with CAD and LEPAD, 16 patients were excluded due to urgent PCI (n = 9), absence of complete laboratory assessment (n = 6), and deferred CAD and LEPAD revascularization for more than 1 year (n = 1). The overall cohort of 135 patients was divided into two groups according to revascularization timing: 45 patients (33.3%) underwent PCI and PTA in the same procedure (concomitant group), whereas 90 patients (66.6%) underwent PCI and PTA in two different procedures (deferred group). Concomitant or deferred CAD and LEPAD percutaneous revascularization strategy was performed according to operator discretion. Study flowchart is represented in Figure 1.

Baseline characteristics of the study population are reported in Table 2. The two groups showed similar baseline characteristics, including age, sex, and body mass index. Except for the familiar history of CAD being more frequent in the concomitant group (17.8% vs. 35.6%, *p* = 0.04), no differences were found in the baseline CV risk profile among study groups. Patients with histories of diabetes mellitus resulted in 66.7% and 54.4% in the concomitant and deferred groups, respectively (*p* = 0.2). Moreover, hypertension (88.9% and 85.6%, *p* = 0.79) and hypercholesterolemia (82.2% and 81.1%, *p* = 1.0) showed similar rates in the concomitant and deferred groups respectively. Even previous MI showed no significant difference considering concomitant vs. deferred group (24.4% vs. 30.0%, *p* = 0.55) while history of CKD was detected in 33.3% and 27.8% among concomitant and deferred groups respectively (*p* = 0.55). Previous history of bleeding (according to thrombolysis in myocardial infarction classification) [14] was similar between groups irrespective of major (2.2% vs. 4.4%, *p* = 0.66) or minor (4.4% vs. 1.1%, *p* = 0.26) bleeding events comparing concomitant vs. deferred groups, respectively. Finally, no difference was found among baseline medications considering ASCVD drugs as antithrombotic, hypertension medications, and lipid-lowering therapy (Table 3). Insulin therapy was more likely prescribed among the concomitant group compared to the deferred group (44.4% vs. 24.4%, *p* = 0.03), while proton pump inhibitor prescriptions were more frequent in the deferred group (51.1% vs. 76.7%, *p* = 0.003).

### 3.2. Coronary and Peripheral Periprocedural Features

Patients in the deferred group presented with complex CAD considering total lesions, the number of diseased vessels, as well as bifurcation lesions. The overall average number of coronary lesions treated was 1.35 ± 0.7, with a higher number of lesions treated in the deferred group (1.5 ± 0.8 vs. 1.1 ± 0.3, *p* < 0.001), where multivessel coronary disease (75.6% vs. 57.8%, *p* = 0.05) and type B2/C lesions according to the ACC/AHA classification were more frequent. In the concomitant group, one lesion was more frequently treated when compared to deferred (88.9% vs. 64.4%, *p* = 0.002), with PCI localized in 95.6% of cases in only one vessel. The treatment of bifurcation lesions was predominant in the deferred group (25.6% vs. 6.7%, *p* = 0.01) as well as length of stents (Table 4). None of the patients in the concomitant group underwent treatment of coronary total occlusion (CTO). During coronary intervention, the femoral artery was the most used vascular access in the concomitant group, while in the deferred group the radial access was mostly prevalent (Table 4). Among peripheral angiographic findings, the number in average of lesions treated was 2 ± 0.8 overall with a similar distribution into two groups. Lesions were mostly localized in the femoral-popliteal site (68.9% of the patients). The prevalence of peripheral CTO was more frequent in the deferred group (35.6% vs. 8.9%, *p* = 0.001). Also, regarding peripheral angioplasty, the lesions treated in the deferred group were significantly longer, more stenotic, and required more often a stent implantation (Table 5).

The contrast amount was higher in the concomitant group (293.2 ± 100.3 mL vs. 211 ± 92.6 mL, *p* = 0.01). Regarding procedural medications, patients underwent concomitant revascularization and received a higher dose of intravenous unfractioned heparin, as well as more often oral loading doses of P2Y12 inhibitors and intravenous doses of aspirin (Table 4).

### 3.3. Laboratory Assessment

Cardiac troponin I (cTnI), creatine-kinase binding protein (CK-MB), creatinine, and complete hematological evaluation were systematically collected before the index procedure and during the hospitalization. No significant difference was found in myocardial injury incidence between groups. Also, baseline creatinine value (1.42 ± 1.36 mg/dL vs. 1.42 ± 1.50 in concomitant and deferred groups, respectively; *p* = 0.98) and hemoglobin value (12.66 ± 1.72 g/dL vs. 13.05 ± 1.86 g/dL in concomitant and deferred groups, respectively; *p* = 0.23) were comparable among study groups.

After 24 h from PCI, the cTnI peak increased in the deferred group (4655.23 ± 26,074.78 ng/L) vs. the concomitant group (568.91 ± 971.64 ng/L, *p* = 0.0005). CK-MB peak resulted in lower concomitant (4.47 ± 4.09 UI/L) vs. deferred group (11.81 ± 37.06 UI/L) without statistical significance (*p* = 0.07). Finally, hemoglobin and creatinine assessments found no difference among groups (Table 6).

### 3.4. Clinical Outcomes

In-hospital clinical outcomes are presented in Table 7. Periprocedural MI occurred more frequently in the deferred group (4.4% vs. 18.9%, *p* = 0.04 in the concomitant and deferred groups, respectively). The occurrence of myocardial injury (77.8% vs. 62.0%, *p* = 0.07) and CIN (11.1% vs. 8.9%, *p* = 0.68) were similar between two study groups. Despite the more frequent adoption of the femoral artery approach in the concomitant group, no significant differences were found in vascular access complications (*p* = 0.21). Considering in hospital CIN, no significant differences were found among the concomitant (11.1%) vs. deferred (8.9%) group, OR 1.3; 95% CI 0.36–4.21.

**Figure 2:**
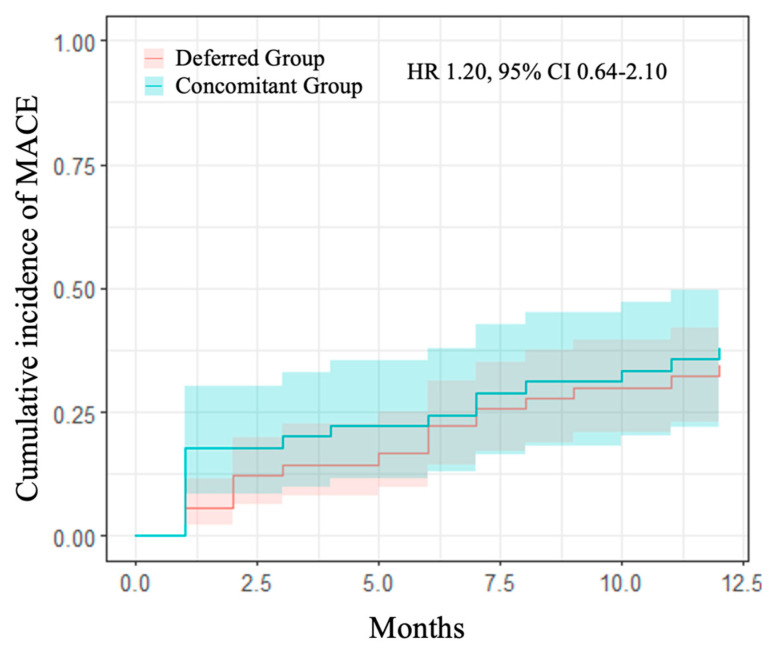
1-year cumulative incidence of MACE comparing the study groups. Kaplan–Meier curves for primary efficacy endpoint (MACE—a composite of spontaneous MI, death for all causes, any PTA, any PCI, any amputation for vascular causes, and ischemic stroke). In blue, concomitant group vs. separate group in red with relative confidence intervals. HR: hazards ratio; CI: confidence interval.

At 1 year follow-up, no differences were detected on the primary efficacy endpoint of MACE among the two groups (37.8% and 34.4%, HR 1.20, 95% CI 0.64–2.10, in the concomitant and deferred groups, respectively) (Table 8, Figure 2). Moreover, 1-year MALE occurrence was similar between study groups (24.4% vs. 27.8% in concomitant and deferred groups, respectively), *p* = 0.77.

Even the incidence of the single adverse events after one month did not show any significant difference. Considering concomitant vs. deferred group, all-causes of death occurred in 0% vs. 1.1% (*p* = 1), spontaneous MI in 4.4% vs. 1.1% (*p* = 0.25), any-PTA in 4.4% vs. 5.6% (*p* = 0.79) with no cases of ischemic stroke. Patients in the concomitant group had more frequently amputation adverse at 1 month follow-up (6.7%) compared to the deferred group (0%), *p* = 0.03, as well as any PCI in the concomitant (8.9%) vs. the deferred group (0%), *p* = 0.01 (Table 8).

After 1-year of follow-up, no differences among two study groups were found in all-causes death (6.7% in concomitant and 2.2% in deferred, *p* = 0.21), MI (4.4% vs. 3.3%, *p* = 0.74), any-PTA (20.0% vs. 27.8%, *p* = 0.31) and any PCI (8.9% vs. 7.8%, *p* = 0.79) without any IS. Even at 1-year follow-up, amputation rates increased in the concomitant vs. deferred group (6.7% vs. 0%, *p* = 0.03) (Table 8).

## 4. Discussion

This study is focused on the evaluation of the safety and efficacy of concomitant versus deferred CAD and PAD revascularization in patients affected by MVD, representing a high CV risk population for hard clinical endpoint [15].

Among PAD patients, prior evidence confirmed the risk of MACE, with 20% of patients with intermittent claudication experiencing MI or stroke at 5 years follow-up, resulting in a related mortality of 10–15% [14]. Conversely, CAD patients with concomitant LEPAD exhibited a 2-fold increased rate of all-cause death and MACE in the PEGASUS TIMI-54 trial [16]. Our population’s baseline risk profile aligns with other clinical trials enrolling similar risk-profiles [9,13].

Managing percutaneous revascularization timing among CAD and LEPAD patients poses a challenge. To date, only one study investigated the timing of coronary and peripheral revascularization within a 12 month frame, showing no differences in the incidence of MACE, mortality, and CIN [17]. Our study demonstrates the safety and efficacy of concomitant CAD and LEPAD percutaneous revascularization among a very high CV risk population. Considering the primary efficacy endpoint, 1 year rates of MACE were similar between study groups, suggesting that a strategy of concomitant revascularization is effective and safe, particularly for patients with non-complex CAD. The prognostic value of MACE among these patients was assessed by the TRA 2°P-TIMI 50 trial, revealing an almost twofold higher rate of MACE at 3 years in patients with MVD compared to those with only peripheral disease (12.8% vs. 7.6%) [18]. In our study, even other 1-year efficacy endpoints were comparable in both groups, but we observed a higher rate of amputation and any PCI in the concomitant group. We may speculate that concomitant revascularization enhances patient compliance due to a single hospitalization and procedure, thereby reducing inherent costs. Some concerns may arise regarding the widespread use of femoral access. Given the complexity of concomitant coronary and lower extremity revascularization, in some cases operators preferred femoral access considering complex procedures.

Despite strong evidence supporting radial artery access as the preferred route for PCI [19], our study did not find a significant difference between the two approaches. This might be attributed to the local expertise in peripheral and structural intervention. The rate of complications associated with femoral access, detected through ultrasound, was as high as 8.9%, with no statistical variances between the two-study group, consistent with other reports [20].

Considering the characteristics of the coronary lesions, we observed higher complexity, total lesion count, number of diseased vessels, and bifurcation lesions in the deferred group, with no left main lesions treated in the concomitant group. This observation may be a consequence of operators’ choice to manage peripheral and coronary revascularization separately upon encountering complex CAD. Similarly, in the deferred group, we found a higher complexity of lower extremity arterial disease (longer lesions, higher degree of stenosis, presence of CTO). Contrast-induced nephropathy is a common complication after percutaneous procedures, correlated with the amount of contrast medium used and pre-existing CKD as the strongest predictor [21]. Previous studies confirmed CIN impact on renal function and on long-term prognosis, particularly when kidney injury is persistent [22]. Our center employs a preventive protocol, including hydration with a saline solution of NaCl 0.9% at 1 mL/kg/h starting 12 h before the procedure and continuing until 12 h after, with an adjustment rate according to the patient’s ejection fraction, filling pressures, and volemic state [20]. Despite the mean amount of contrast medium being higher in the concomitant group, CIN occurrence was comparable between the concomitant and deferred groups, emphasizing the importance of adopting the preventive protocol. Another adverse event affecting the prognosis of patients undergoing PCI for stable CAD is periprocedural MI [23]. In our study, we found an overall incidence of periprocedural MI of 14.1%, driven by the deferred group (18.9%) and aligned with previously reported data [23,24]. Moreover, in these registries, patients affected by periprocedural MI had a higher complex degree of coronary disease [25]. Accordingly, in our study, periprocedural MI incidence was significantly higher in the deferred group, which exhibited more complex CAD with multivessel complex lesions leading to periprocedural MI.

In summary, the right revascularization timing between coronary and lower extremity peripheral artery disease is still debated. There is a lack of data in this field since the challenge to design a dedicated randomized trial on clinical outcomes comparing concomitant vs. deferred coronary and lower extremity revascularization. The most important barrier may be the systematic selection of patients suitable for elective revascularization procedures, even considering the different approach (i.e., percutaneous, surgical, or hybrid). Moreover, compared to coronary, LEPAD percutaneous revascularization still shows wide variability in terms of operators and lesions management.

## 5. Limitations

Our study has several limitations. First, it is an observational single-center retrospective study based on a small sample size with no formal assessment performed. The study reference period is as long as 9 years, a range of time with extensive changes in catheterization laboratory equipment, indications for revascularization, and medical management. Finally, management of CAD/LEPAD revascularization in either concomitant or deferred procedures was according to operators’ choice and not based on systematic stratification.

## 6. Conclusions

Concomitant CAD and PAD percutaneous revascularization results are effective and safe regarding the occurrence of MACE at 1 year follow-up and in hospital CIN in a very high risk population with multisite vascular disease. Dedicated studies are warranted to confirm short and long-term outcomes of concomitant CAD and LEPAD revascularization.

## Figures and Tables

**Table 1:** Study inclusion and exclusion criteria.

Inclusion Criteria	Exclusion Criteria
1. Patients > 18 years old.	1. Patient who did not sign the informed consent.
2. Patients affected by LEAD and CAD underwent coronary and peripheral percutaneous revascularization.	2. Patient with not-significant coronary or peripheral artery disease.
	3. Patients underwent primary or urgent PCI.
	4. Patients underwent urgent PTA.
	5. Pregnant woman.
	6. Period of deferred percutaneous intervention > 1 year.

CAD: coronary artery disease; LEPAD: lower extremity peripheral artery disease; PCI: percutaneous coronary intervention; PTA: percutaneous transluminal angioplasty.

**Table 2:** Demographic baseline characteristics of the study population.

	Total Population n = 135	Concomitant Group n = 45	Deferred Group n = 90	*p* Value
**Age, years**	66 ± 10	65.3 ± 10.1	66.6 ± 9.4	0.48
**Female sex**	23 (17)	10 (22.2)	13 (14.4)	0.33
**Height (cm)**	170 ± 8	168.9 ± 9	169.8 ± 7.4	0.58
**Weight (kg)**	78 ± 13	77.1 ± 12.6	78.4 ± 13.5	0.57
BMI *	27 ± 4	26.9 ± 4	27.1 ± 4.4	0.82
**Family history of CAD**	40 (29.6)	8 (17.8)	32 (35.6)	0.04
**Diabetes Mellitus**	79 (58.5)	30 (66.7)	49 (54.4)	0.20
** Oral treatment**	30 (38.0)	10 (33.3)	20 (40.8)	0.63
** Insulin dependent**	47 (59.5)	20 (66.7)	27 (55.1)	0.35
** Diet**	2 (2.5)	0 (0.0)	2 (4.1)	0.52
**Smoking**				
** Non smoker**	21 (15.6)	6 (13.3)	15 (16.7)	0.80
** Current**	53 (39.3)	16 (35.6)	37 (41.1)	0.58
** Former**	61 (45.2)	23 (51.1)	38 (42.2)	0.36
**Hypertension**	117 (86.7)	40 (88.9)	77 (85.6)	0.79
**Hypercholesterolemia**	110 (81.5)	36 (82.2)	73 (81.1)	1
**Previous Myocardial Infarction**	38 (28.1)	11 (24.4)	27 (30.0)	0.55
**Left Ventricular Ejection Fraction †**	54 ± 9	54.6 ± 6.5	53.2 ± 9.9	0.33
**Congestive Heart Failure**	18 (13.3)	5 (11.1)	13 (14.4)	0.79
**Previous PCI**	51 (37.8)	16 (35.6)	35 (38.9)	0.85
**Previous CABG**	10 (7.4)	1 (2.2)	9 (10.0)	0.16
**CKD (eGFR ‡ < 60 mL/min)**	40 (29.6)	14 (33.3)	25 (27.8)	0.55
**Chronic Obstructive Pulmonary Disease**	33 (24.4)	8 (17.8)	25 (27.8)	0.29
**Anemia**	63 (46.7)	23 (51.1)	40 (44.4)	0.47
**Prior Stroke or TIA**	13 (9.6)	5 (11.1)	8 (8.9)	0.76
**Hystory of AF/atrial flutter**	8 (5.9)	3 (6.7)	5 (5.6)	1
**History of TIMI bleeding**	8 (5.9)	3 (6.7)	5 (5.6)	1
** TIMI Major bleeding**	5 (3.7)	1 (2.2)	4 (4.4)	0.66
** TIMI Minor bleeding**	3 (2.2)	2 (4.4)	1 (1.1)	0.26

Data expressed as n (%) or means ± SD. *p*-values from Fisher’s tests or χ2 tests or unpaired *t*-tests. AF: atrial fibrillation; BMI: body mass index; CABG: coronary artery bypass grafting; CAD: coronary artery disease; CKD: chronic kidney disease; eGFR: estimated glomerular filtration rate; PCI: percutaneous coronary intervention; SD: standard deviation; TIA: transient ischemic attack; TIMI: thrombolysis in myocardial infarction. * The body-mass index is the weight in kilograms divided by the square of the height in meters. † LVEF is calculated with 2D echocardiography as equation (end-diastolic volume–end-systolic volume)/(end-diastolic volume) with Simpson’s biplane method. ‡ eGFR is calculated using MDRD 4-variable equation.

**Table 3:** Medications at baseline.

	Total Population n = 135	Concomitant Group n = 45	Deferred Group n = 90	*p* Value
**Aspirin**	104 (77.0)	31 (68.9)	73 (81.1)	0.13
**P2Y12 inhibitors**				
** None**	51 (37.8)	21 (46.7)	30 (33.3)	0.14
** Clopidogrel**	77 (57.0)	24 (53.3)	53 (58.9)	0.58
** Ticagrelor**	7 (5.2)	0 (0.0)	7 (7.8)	0.09
**Beta Blockers**	75 (55.6)	25 (55.6)	50 (55.6)	1
**Statin**	109 (80.7)	33 (73.3)	76 (84.4)	0.16
**Other lipid lowering drug**	14 (10.4)	5 (11.1)	9 (10.0)	1
**Calcium channel blockers**	47 (34.8)	17 (37.8)	30 (33.3)	0.70
**ACE/ARB inhibitors**	92 (68.1)	33 (73.3)	59 (65.6)	0.44
**Sacubitril/Valsartan**	1 (0.7)	0 (0.0)	1 (1.1)	1
**Diuretics**	44 (32.6)	14 (31.1)	30 (33.3)	0.85
**Oral Anti-Coagulation**				
** None**	130 (96.3)	44 (97.8)	85 (95.6)	0.66
** DOAC**	4 (3.0)	1 (2.2)	3 (3.3)	1
** VKA**	1 (0.7)	0 (0.0)	1 (1.1)	1
**Oral antidiabetic drugs**	42 (31.1)	15 (33.3)	27 (30.0)	0.70
**Insulin**	42 (31.1)	20 (44.4)	22 (24.4)	0.03
**Pump Proton Inhibitor**	92 (68.1)	23 (51.1)	69 (76.7)	0.003

Data expressed as n (%) or means ± SD. *p*-values from Fisher’s tests or χ2 tests or unpaired *t*-tests. ACE: angiotensin-converting enzyme; ARB: angiotensin receptor blockers; DOAC: direct oral anti-coagulant; SD: standard deviation; VKA: vitamin K antagonist.

**Table 4:** Coronary angiography findings.

	Total Population n = 135	Concomitant Group n = 45	Deferred Group n = 90	*p* Value
**Number of treated lesions**	1.35 ± 0.7	1.1 ± 0.3	1.5 ± 0.8	<0.0001
** 1**	98 (72.6)	40 (88.9)	58 (64.4)	0.002
** 2**	30 (22.2)	5 (11.1)	25 (27.8)	0.03
** 3**	5 (3.7)	0 (0.0)	5 (5.6)	0.17
** 4**	1 (0.7)	0 (0.0)	1 (1.1)	1
** 5**	1 (0.7)	0 (0.0)	1 (1.1)	1
**Multivessel disease**	94 (69.6)	26 (57.8)	68 (75.6)	0.05
**Target lesion**				
** RCA**	49 (36.3)	13 (28.9)	36 (40.0)	0.26
** LAD**	70 (51.9)	27 (60.0)	43 (47.8)	0.20
** LCx**	32 (23.7)	7 (15.6)	25 (27.8)	0.14
** LM,**	8 (5.9)	0 (0.0)	8 (8.9)	0.05
**3 vessel treated**	3 (2.2)	0 (0.0)	3 (3.3)	0.55
**2 vessel treated**	21 (15.6)	2 (4.4)	19 (21.1)	0.01
**1 vessel treated**	112 (83.0)	43 (95.6)	69 (76.7)	0.01
**At least one restenotic lesion**	22 (16.3)	5 (11.1)	17 (18.9)	0.33
At least one type B2/C lesion *	50 (37.0)	14 (31.1)	36 (40.0)	0.35
**≥3 stent implanted**	22 (16.3)	2 (4.4)	20 (22.2)	0.01
**Total stent length**	35 ± 22.9	27.6 ± 14.9	38.7 ± 25.2	0.00
**Invasive imaging assessment**				
**None**	121 (89.6)	41 (91.1)	80 (88.9)	0.77
**IVUS**	8 (5.9)	2 (4.4)	6 (6.7)	0.72
**OCT**	6 (4.4)	2 (4.4)	4 (4.4)	1
**Invasive assessment of ischemia †**	21 (15.6)	9 (20.0)	12 (13.3)	0.32
**Periprocedural Medications**				
** Aspirin load**	34 (25.2)	19 (42.2)	15 (16.7)	0.003
** Clopidogrel**	72 (53.3)	37 (82.2)	35 (38.9)	0.002
** Loading dose**				
** None**	66 (48.9)	10 (22.2)	56 (62.2)	0.01
** 300 mg**	32 (23.7)	16 (35.6)	16 (17.8)	0.03
** 600 mg**	37 (27.4)	19 (42.2)	18 (20.0)	0.01
** Cangrelor**	3 (2.2)	0 (0.0)	3 (3.3)	0.55
** ** **UFH dose (IU)**	5659.3 ± 1408.5	6322.2 ± 1696.1	5327.8 ± 1109.6	<0.0001
** ** **GP IIb/IIIa Inhibitor (Tirofiban)**	1 (0.7)	0 (0.0)	1 (1.1)	1
**Access site**				
**Femoral**	90 (66.7)	43 (95.6)	47 (52.2)	<0.0001
**Radial**	45 (33.3)	2 (4.4)	43 (47.8)	<0.0001
**Treatment of heavily calcified lesion**	18 (13.3)	3 (6.7)	15 (16.7)	0.18
** Rotational atherectomy**	0 (0.0)	0 (0.0)	0 (0.0)	1
** Shockwave**	1 (0.7)	0 (0.0)	1 (1.1)	1
**Chronic total occlusion**	5 (3.7)	0 (0.0)	5 (5.6)	0.17
**Treatment of bifurcation lesion**	26 (19.3)	3 (6.7)	23 (25.6)	0.01
** Bifurcation with 2 stents**	7 (5.2)	0 (0.0)	7 (7.8)	0.09

Data expressed as n (%) or means ± SD. *p*-values from Fisher’s tests or χ2 tests or unpaired *t*-tests. IU: international unit; IVUS: intravascular ultrasound; LAD: left anterior descending; LCx: left circumflex; LM: left main; OCT: optical coherence tomography; RCA: right coronary artery; UFH: unfractioned heparin. * Coronary lesions classification according to ACC/AHA classification. [† Invasive assessment of ischemia is considered a value of fractional flow reserve (FFR) < 0.80.

**Table 5:** Peripheral angiography findings.

	Total Population n = 135	Concomitant Group n = 45	Deferred Group n = 90	*p* Value
**Femoral access**	134 (99.3)	44 (97.8)	90 (100.0)	0.33
**Bilateral femoral access**	25 (18.5)	12 (26.7)	13 (14.4)	0.1
**Radial access**	9 (6.7)	6 (13.3)	3 (3.3)	0.06
**Sheath size (Fr)**	5.7 ± 0.7	5.7 ± 0.8	5.7 ± 0.7	0.62
**Number of lesions**	2 ± 0.8	1.4 ± 0.8	1.4 ± 0.6	0.93
** 1**	93 (68.9)	32 (71.1)	61 (67.8)	0.84
** 2**	34 (25.2)	10 (22.2)	24 (26.7)	0.68
** 3**	6 (4.4)	2 (4.4)	4 (4.4)	1
** 4**	1 (0.7)	0 (0.0)	1 (1.1)	1
** 5**	1 (0.7)	1 (2.2)	0 (0.0)	0.33
**Aorto-iliac lesion**	36 (26.7)	11 (24.4)	25 (27.8)	0.84
**Femoro-popliteal lesion**	93 (68.9)	28 (62.2)	65 (72.2)	0.24
**Below the knee lesion**	29 (21.5)	12 (26.7)	17 (18.9)	0.37
**Restenotic lesion**	4 (3.0)	3 (6.7)	1 (1.1)	0.11
**Chronic total occlusion**	36 (26.7)	4 (8.9)	32 (35.6)	0.001
**Thrombus**	3 (2.2)	0 (0.0)	3 (3.3)	0.55
**Severe calcification**	11 (8.1)	4 (8.9)	7 (7.8)	1
**Target lesion lenght (mm)**	62.5 ± 51.2	48 ± 30	70 ± 58.1	0.002
**Reference vessel diameter (mm)**	5 ± 1.7	5 ± 1.9	5 ± 1.6	0.90
**MLD (mm) before PTA**	1.2 ± 1.2	1.4 ± 1	1.1 ± 1.3	0.10
**MLD (mm) after PTA**	3.8 ± 1.4	3.7 ± 1.4	3.9 ± 1.4	0.44
**Target lesion stenosis**	86.3 ± 14.4	82.5 ± 13.9	88.2 ± 14.4	0.01
**Pre-dilatation**	40 (24.2)	7 (12.7)	33 (30.0)	0.01
**POBA**	80 (59.3)	28 (62.2)	52 (57.8)	0.71
**Drug Eluting Balloon**	48 (35.6)	16 (35.6)	32 (35.6)	1
**Drug Eluting Stent**	17 (12.6)	2 (4.4)	15 (16.7)	0.05
**Bare Metal Stent**	31 (23.0)	11 (24.4)	20 (22.2)	0.83
**Post-dilatation**	36 (26.7)	10 (22.2)	26 (28.9)	0.42
**Maximum device diameter (mm)**	5.2 ± 1.5	4.8 ± 1.5	5.4 ± 1.4	0.04
**Maximum device length (mm)**	62.6 ± 52.4	40 ± 32	74 ± 57.1	0.02
**Thrombus aspiration**	2 (1.5)	0 (0.0)	2 (2.2)	0.55
**Atherectomy**	1 (0.7)	1 (2.2)	0 (0.0)	0.33
**Post-procedural dissection**	13 (9.6)	3 (6.7)	10 (11.1)	0.54
Dissection type *				
** Dissection type AC**	7 (58.3)	2 (66.7)	5 (55.6)	1
** Dissection type DF**	6 (50.0)	1 (33.3)	5 (55.6)	1
**Procedural success**	133 (98.5)	45 (100.0)	88 (97.8)	0.55
**Contrast medium (mL)**	238.4 ± 102.6	293.2 ± 100.3	211 ± 92.6	0.01
**Procedure time (min)**	79.8 ± 42.5	86.1 ± 36	76.6 ± 45.2	0.19
**Fluoroscopy time (min)**	29.4 ± 17.9	33.2 ± 19.5	27.6 ± 16.8	0.1
**Femoral manual compression**	85 (63.9)	32 (71.1)	53 (60.2)	0.25
**Femoral access closure device**				
** Proglide**	13 (9.8)	4 (8.9)	9 (10.2)	1
** AngioSeal**	38 (28.6)	9 (20.0)	29 (33.0)	0.16

Data expressed as n (%) or means ± SD. *p*-values from Fisher’s tests or χ2 tests or unpaired *t*-tests. MLD: minimal lumen diameter; POBA: plain old balloon angioplasty. * Classification of post-procedural dissection.

**Table 6:** Laboratory findings.

	Total Population n = 135	Concomitant Group n = 45	Deferred Group n = 90	*p* Value
**Pre-PCI**				
cTn-I (ng/L) *	12.92 ± 16.38	14.63 ± 19.47	12.07 ± 14.64	0.44
**CK-MB (ng/mL) †**	2.77 ± 4.20	2.46 ± 1.67	2.92 ± 5.01	0.43
**Creatinine (g/dL)**	1.42 ± 1.45	1.42 ± 1.36	1.42 ± 1.50	0.98
**Hb (g/dL)**	12.92 ± 1.81	12.66 ± 1.72	13.05 ± 1.86	0.23
**24 h after PCI**				
**cTn-I peak (ng/L)**	3293.12 ± 21,345.25	568.91 ± 971.64	4655.23 ± 26,074.78	0.0005
**CK-MB peak (ng/mL)**	9.36 ± 30.49	4.47 ± 4.09	11.81 ± 37.06	0.07
**Creatinine peak (mg/dL)**	1.53 ± 1.45	1.58 ± 1.38	1.51 ± 1.49	0.80
**Hb (g/dL)**	11.90 ± 1.85	11.27 ± 1.78	12.21 ± 1.81	0.004

CK-MB: creatine kinase-myoglobin binding; cTn-I: cardiac troponin I; Hb: hemoglobin; PCI: percutaneous coronary intervention; URL: upper reference limit. * Hs-cTn I was measured using the Abbott ARCHITECT STAT High Sensitive Troponin-I assay (Abbott Laboratories, Chicago, IL, USA), which has a lower limit of detection of 1.2 ng/L and a 99th percentile URL of 34 ng/L in men and 16 ng/L in women. † CK-MB was measured using the Abbott ARCHITECT STAT CK-MB assay (Abbott Laboratories, Chicago, IL, USA), which has a lower limit of detection of 1.5 ng/mL and a 99th percentile URL of 7.2 ng/mL in men and 3.4 ng/mL in women.

**Table 7:** In hospital outcomes.

	Total Population n = 135	Concomitant PCI/PTA n = 45	Deferred PCI/PTA n = 90	*p* Value
**Cardiac arrest**	0 (0.0)	0 (0.0)	0 (0.0)	1
Periprocedural MI *	19 (14.1)	2 (4.4)	17 (18.9)	0.04
Myocardial injury *	91 (67.4)	35 (77.8)	56 (62.2)	0.07
Contrast Induced Nephropathy *	13 (9.6)	5 (11.1)	8 (8.9)	0.68
**Vascular Access Complications**	12 (8.9)	6 (13.3)	6 (6.7)	0.21

Data expressed as n (%). *p* values from logistic regression model. MI: myocardial infarction. * See the text to find out definitions of periprocedural MI, myocardial injury, and CIN.

**Table 8:** Follow up after 1 month and 1 year.

	Total Population n = 135	Concomitant Group n = 45	Deferred Group n = 90	*p* Value
**One month**				
** ** **All-cause death**	1 (0.7)	0 (0.0)	1 (1.1)	1
** Spontaneous MI**	3 (2.2)	2 (4.4)	1 (1.1)	0.25
** Any PTA**	7 (5.2)	2 (4.4)	5 (5.6)	0.79
** Amputation**	3 (2.2)	3 (6.7)	0 (0.0)	0.03
** Any PCI**	4 (3.0)	4 (8.9)	0 (0.0)	0.01
** Ischemic Stroke**	0 (0.0)	0 (0.0)	0 (0.0)	1
** ** **MALE**	10 (7.4)	5 (11.1)	5 (5.6)	0.22
** MACE**	15 (11.1)	9 (20.0)	6 (6.7)	0.02
**One year**				
** All-cause death**	5 (3.7)	3 (6.7)	2 (2.2)	0.21
** Spontaneous MI**	5 (3.7)	2 (4.4)	3 (3.3)	0.74
** Any PTA**	34 (25.2)	9 (20.0)	25 (27.8)	0.31
** Amputation**	3 (2.2)	3 (6.7)	0 (0.0)	0.03
** Any PCI**	11 (8.1)	4 (8.9)	7 (7.8)	0.79
** Ischemic Stroke**	0 (0.0)	0 (0.0)	0 (0.0)	1
** MALE**	36 (26.7)	11 (24.4)	25 (27.8)	0.77
** MACE**	48 (35.6)	17 (37.8)	31 (34.4)	0.61

Data expressed as n (%). *p* values from Cox’s proportional hazard models. FU: follow-up; MACE: major adverse cardiovascular events; MALE: major adverse limb events; MI: myocardial infarction; PCI: percutaneous coronary intervention; PTA: percutaneous transluminal angioplasty.

## Data Availability

Data available upon reasonable request to corresponding author.

## Abbreviations

ACC	American College of Cardiology
AHA	American Heart Association
ASCVD	atherosclerotic cardiovascular disease
CAD	coronary artery disease
CKD	chronic kidney disease
CI	confidence intervals
CIN	contrast induced nephropathy
CTO	chronic total occlusion
CV	cardiovascular
DM	diabetes mellitus
eGFR	estimated glomerulation filtration rate
ESC	European Society of Cardiology
LEPAD	lower extremity peripheral artery disease
LER	lower extremity revascularization
MACE	major adverse cardiovascular events
MALE	major adverse limb events
MI	myocardial infarction
MVD	multisite vascular disease
PCI	percutaneous coronary intervention
PTA	percutaneous transluminal angioplasty
UFH	unfractionated heparin
